# The dual role of glucocorticoid regeneration in inflammation at parturition

**DOI:** 10.3389/fimmu.2024.1459489

**Published:** 2024-09-03

**Authors:** Li-Jun Ling, Qiong Zhou, Fan Zhang, Wen-Jia Lei, Meng-Die Li, Jiang-Wen Lu, Wang-Sheng Wang, Kang Sun, Hao Ying

**Affiliations:** ^1^ Department of Obstetrics, Shanghai First Maternity and Infant Hospital, School of Medicine, Tongji University, Shanghai, China; ^2^ Shanghai Key Laboratory of Maternal Fetal Medicine, Shanghai Institute of Maternal-Fetal Medicine and Gynecologic Oncology, Shanghai, China; ^3^ Department of Obstetrics and Gynecology, Ren Ji Hospital, Shanghai Jiao Tong University School of Medicine, Shanghai, China; ^4^ Center for Reproductive Medicine, Ren Ji Hospital, Shanghai Jiao Tong University School of Medicine, Shanghai, China; ^5^ Shanghai Key Laboratory for Assisted Reproduction and Reproductive Genetics, Shanghai, China

**Keywords:** glucocorticoid, fetal membranes, chorioamnionitis, 11β-HSD1, preterm birth

## Abstract

**Introduction:**

Fetal membrane inflammation is an integral event of parturition. However, excessive pro-inflammatory cytokines can impose threats to the fetus. Coincidentally, the fetal membranes express abundant 11β-hydroxysteroid dehydrogenase 1 (11β-HSD1), which generates biologically active cortisol to promote labor through induction of prostaglandin synthesis. Given the well-recognized anti-inflammatory actions of glucocorticoids, we hypothesized that cortisol regenerated in the fetal membranes might be engaged in restraining fetus-hazardous pro-inflammatory cytokine production for the safety of the fetus, while reserving pro-labor effect on prostaglandin synthesis to ensure safe delivery of the fetus.

**Methods:**

The hypothesis was examined in human amnion tissue and cultured primary human amnion fibroblasts as well as a mouse model.

**Results:**

11β-HSD1 was significantly increased in the human amnion in infection-induced preterm birth. Studies in human amnion fibroblasts showed that lipopolysaccharide (LPS) induced 11β-HSD1 expression synergistically with cortisol. Cortisol completely blocked NF-κB-mediated pro-inflammatory cytokine expression by LPS, but STAT3-mediated cyclooxygenase 2 expression, a crucial prostaglandin synthetic enzyme, remained. Further studies in pregnant mice showed that corticosterone did not delay LPS-induced preterm birth, but alleviated LPS-induced fetal organ damages, along with increased 11β-HSD1, cyclooxygenase 2, and decreased pro-inflammatory cytokine in the fetal membranes.

**Discussion:**

There is a feed-forward cortisol regeneration in the fetal membranes in infection, and cortisol regenerated restrains pro-inflammatory cytokine expression, while reserves pro-labor effect on prostaglandin synthesis. This dual role of cortisol regeneration can prevent excessive pro-inflammatory cytokine production, while ensure in-time delivery for the safety of the fetus.

## Introduction

Inflammation of the fetal membranes plays a crucial role in labor onset in both term and preterm by inducing a wealth of labor-inducing factors including prostaglandins, matrix metalloproteases and pro-inflammatory cytokines etc. (1–3). Based on the presence or absence of infection, inflammation of the fetal membranes can be classified into infectious and sterile inflammation. Although sterile inflammation of the fetal membranes is an integral event of normal parturition, uncontrolled inflammation, particularly in infection-induced chorioamnionitis, can lead to preterm birth by producing excessive pro-inflammatory cytokines (4, 5). In addition to induction of labor, excessive pro-inflammatory cytokines can also pose fatal threats to the fetus by developing fetal inflammatory response syndrome (FIRS), a condition of fetal systemic inflammation with rapid elevation of pro-inflammatory cytokines (such as IL-1β and IL-6) and leucocytes in the fetal circulation (6, 7). FIRS can lead to multiple fetal organ damages, neonatal sepsis, and even fetal death (8–11). Therefore, it is of utmost importance to balance inflammation reactions of the fetal membranes precisely to allow the baby to be delivered safely. Here we examined whether there exists such a mechanism to balance the inflammatory reactions of the fetal membranes in normal and infection-induced parturition.

Glucocorticoids (GCs) are a class of steroid hormones which are recognized to have powerful anti-inflammatory actions with inhibition of nuclear factor-κB (NF-κB) as one of the central mechanisms (12). The adrenal cortex is known to be the primary site of *de novo* synthesis of glucocorticoids (13). In addition to the *de novo* synthesis in adrenal glands, there also exists an alternative glucocorticoid-regenerating pathway in virtually all glucocorticoid target tissues (14–17). In these tissues, biologically active glucocorticoids can be regenerated from their biologically inactive keto counterparts by 11β-hydroxysteroid dehydrogenase 1 (11β-HSD1) within the endoplasmic reticulum (18, 19). Specifically, 11β-HSD1 converts biologically inactive cortisone into active cortisol in humans, while converting inert 11-dehydrocorticoserone into active corticosterone in rodents (19). This intracellular glucocorticoid regenerating machinery is believed to be a supplemental gating mechanism to the circulating glucocorticoids to ensure the presence of adequate glucocorticoids for activation of the low affinity glucocorticoid receptor (GR) (14–16).

Notably, the human fetal membranes express abundant 11β-HSD1 and boast the largest glucocorticoid regenerating capacity among fetal tissues (20–22), which increases with gestational age, and further increases at parturition (23, 24). As a result, cortisol concentrations increase in the fetal membranes and amniotic fluid towards the end of gestation, reaching peak levels at parturition (fetal membranes: 6.58 ± 1.61 μM (25, 26), amniotic fluid: 97.66 ± 7.17 nM (27)). Of interest, 11β-HSD1 expression is under the synergistic feedforward induction by glucocorticoids and pro-inflammatory cytokines in the fetal membranes (28–30), indicating that glucocorticoid regeneration capacity may be greatly enhanced by inflammation of the fetal membranes. We and others have demonstrated that one of the prominent roles of glucocorticoid regenerated by 11β-HSD1 is to promote parturition by inducing prostaglandin E2 and F2α synthesis, and remodeling extracellular matrix (ECM) in the amnion layer of the fetal membranes (31–36), the events crucial for labor onset and membrane rupture (24, 26, 37–39). Of interest, a NF-κB-independent pathway has been revealed to mediate the prostaglandin inducing, and ECM remodeling effects of glucocorticoids in the fetal membranes with the involvement of GR interacting with other transcription factors such as STAT3, CREB and C/EBPδ (24, 26, 33).

Given the central role of NF-κB in inflammatory reactions (40), the powerful repressive effect of glucocorticoids on NF-κB (41) and the NF-κB-independent labor-inducing effects of glucocorticoids in the fetal membranes (24, 26, 33), we postulated that glucocorticoids regenerated in the fetal membranes might play a dual role in parturition. On the one hand, glucocorticoids might initiate parturition through NF-κB-independent actions, while restraining inflammatory reactions through NF-κB-dependent actions on the other hand. As such, fetal damage by excessive pro-inflammatory cytokines at parturition could be possibly avoided or ameliorated, particularly in infection-induced chorioamnionitis. As such, boosting glucocorticoid regeneration in the fetal membranes should be considered to increase the chance of fetal survival in infection-induced chorioamnionitis. In this study, we examined the hypothesis by using both cultured human primary amnion fibroblasts, a major cell type synthesizing both pro-labor and pro-inflammatory factors in the fetal membranes, and a mouse model of infection-induced preterm labor, with the ultimate goal to determine whether it is beneficial to enhance glucocorticoid regeneration in the fetal membranes when the fetus is threatened by intrauterine infection.

## Materials and methods

### Collection of the human amnion

The human amnion was collected from either elective cesarean section at term (designated as term none labor, TNL), or spontaneous labor at term without infection (designated as term labor, TL), or preterm labor with infection (designated as preterm labor with infection, PL) with written informed consent under a protocol approved by the ethics committee of Shanghai First Maternal and Infant Hospital, Tongji University School of Medicine. The amnion from the TNL group was used for cell culture. The amnion from the TL and PL groups was snap-frozen in liquid nitrogen for protein extraction. Criteria for determining intrauterine infection in preterm birth included elevated body temperature and white blood cell count, and detection of pathogens in culture of uterine secretion or placental swab. Bacterial culture, antimicrobial susceptibility testing and Gram staining were used for identification of the pathogen.

### Preparation of human amnion fibroblasts

The amnion from TNL was used for isolation of amnion fibroblasts. After delivery, the inner amnion layer was identified at the rupture site of the fetal membranes, and carefully peeled off the chorion layer by hands. The amnion was rapidly transferred to the laboratory in sterile PBS at 4°C within 30 minutes. After thoroughly washing the surface blood of the amnion with sterile PBS, the amnion was digested twice with 0.125% trypsin (#27250-018, Life Technologies Inc., Grand Island, NY) to remove epithelial cells, and further digested with 0.1% Type I collagenase (#C0130, Sigma, St. Louis, MO) to isolate fibroblasts. Isolated fibroblasts were harvested by centrifugation and 1 × 10^6^ cells were plated in a 6-well plate for culture in Dulbecco’s modified Eagle’s medium (DMEM) containing 10% Fetal Bovine Serum (FBS) and antibiotics (Life Technologies Inc) at 37°C in 5% CO2-95% air. This method of cell isolation yields high purity of amnion fibroblast (>95%), which have been verified previously by staining with mesenchymal (vimentin and fibroblast-specific protein 1) and epithelial (cytokeratin-7) markers (35, 42, 43).

### Treatment of human amnion fibroblasts

In this study, primary human amnion mesenchymal cells without any passage were used. Three days after plating, the primary amnion fibroblasts were treated with the following reagents in FBS-free medium. Fibroblasts were treated with cortisol (1 μM; Sigma), lipopolysaccharide (LPS, from *E. coli* serotype 0111:B4, 10 ng/mL; #L2630, Sigma), or combination of cortisol (1 μM) and LPS (10 ng/mL) for 24 hours to investigate the effect on 11β-HSD1, cyclooxygenase-2 (COX-2, the rate-limiting enzyme in prostaglandin synthesis), IκB-α (the NF-κB inhibitory protein) and pro-inflammatory cytokines (IL-1β and IL-6), and for 3 hours to study the effect on the phosphorylation of STAT3 and p65 (a subunit of NF-κB). Cells were treated with cortisone (10 μM; Sigma) with or without 11β-HSD1 inhibitor 10-J (1 μM; Millipore, Billerica, MA) for 24 hours to examine the involvement of 11β-HSD1 in the effect of cortisone. The cells were treated with cortisol and LPS with or without STAT3 antagonist S3I-201 (50 μM; Selleck, Houston, TX) or toll-like receptor-4 (TLR-4) antagonist TAK-242 (10 μM; R&D systems, Minneapolis, MN) or small interference RNA (siRNA)-mediated knockdown of p65 for 24 hours to examine their role in the induction of COX-2 by cortisol and LPS. Cells were transfected with siRNA (50 nM) against *RELA* (encoding p65) (5′-CCACUUUGGUGUUUCAUAAtt-3′) (GenePharma Co., Shanghai, China) or randomly scrambled siRNA with an electroporator (175 V, 5 ms) (Nepa Gene, Chiba, Japan), and were treated with reagents after recovery for 3 days. The knockdown efficiency was 91.5 + 2.6% (**Supplementary Figure 1**). After treatment, cells were harvested for RNA and protein extraction, and the conditioned culture medium was collected for cytokine measurement with a multiplex kit (Bio-Rad, Austin, TX). The treatment regimen of all reagents was based on previous studies (26, 44–48).

### Measurement of cortisol regeneration in human amnion fibroblasts

To measure cortisol regeneration activity of 11β-HSD1 under the induction by cortisol and LPS, amnion fibroblasts were pretreated with cortisol (1 μM) in the presence or absence of LPS (10 ng/mL) for 24 hours. After removal of the treatments by washing, the cells were incubated with cortisone (10 μM), the substrate of 11β-HSD1, for 6 hours. The conditioned culture medium was then collected for cortisol measurement with a cortisol ELISA kit (#500360, Cayman chemical, Ann Arbor, MI).

### Quantitative real-time polymerase chain reaction and western blotting

The abundance of *HSD11B1* (encoding 11β-HSD1), *PTGS2* (encoding COX-2), *IL1B* (encoding IL-1β), *IL6* (encoding IL-6), *NFKBIA* (encoding IκB-α) and *GAPDH* was determined with qRT-PCR. The relative mRNA abundance was quantitated using the 2^-△△Ct^ method. The primer sequences used for qRT-PCR were illustrated in **Supplementary Table 1**. The abundance of 11β-HSD1, IκB-α, COX-2, total p65, phosphorylated p65 at Ser536 (p-p65), total STAT3, phosphorylated STAT3 at Tyr705 (p-STAT3) and GAPDH was determined with Western blotting. The antibody information was detailed in **Supplementary Table 2**. The bands were visualized using a G-BOX iChemi Chemiluminescence Image Capture system (Syngene, Cambridge, U.K.). The ratio of band intensities of 11β-HSD1, IκB-α, COX-2 or total p65, over that of GAPDH was used to indicate the target protein abundance. The ratio of phosphorylated protein over total protein was used to indicate the level of protein phosphorylation. Detailed methods of qRT-PCR and Western blotting have been described previously (49).

### Animal study

Animal study was conducted following a standard protocol for animal care which was approved by Department of Laboratory Animal Science, Tongji University. C57BL/6 mice (Ziyuan, Hangzhou, China) aging from 10 to 13 weeks were mated overnight. The presence of a vaginal plug next morning was counted as gestational day 0.5 (GD 0.5). Pregnant mice were given LPS (from *E. coli* serotype 0111:B4, 20 μg per dam (50)) with or without corticosterone (5 μM) intraperitoneally on GD 16.5. An equal volume of vehicle was given as control. Some mice were allowed to delivery spontaneously and euthanized after observing the pregnancy outcomes. Some mice were sacrificed for collection of amniotic fluid, fetal membranes and fetal organs (lungs and brains). All mice were euthanized by dislocation of cervical vertebrae after administration of anesthetics (Zoletil 50, 50mg/kg, Virbac, Carros, France). The mice were housed and experimented at the Department of Laboratory Animal Science, Tongji University. The fetal lung was stained with hematoxylin-Eosin (HE), and the fetal brain was stained with immunohistochemistry for ionized calcium binding adaptor molecule 1 (Iba1) for examination of injury. The number of alveoli in three visual fields was counted under a 20x objective and averaged, which was used to evaluate the degree of lung injury. Iba1 expression in frozen brain sections was examined by immunohistochemistry as described previously (24). Fetal organs including fetal brains and fetal membranes, and amniotic fluid were frozen in liquid nitrogen for protein extraction for measurements of pro-inflammatory cytokines (IL-1β and IL-6) and Iba1 with enzyme immunoassay kits (Abclonal, Wuhan, China) and Western blotting respectively.

### Statistical analysis

All data are reported as means ± SEM. The number of experiments for human amnion fibroblasts represents cell preparations from different subjects. After normality examination, paired Student’s t-test or one-way ANOVA followed by the Newman-Keuls multiple comparison test was performed where appropriate. Significance was set at P < 0.05.

## Results

### Increased 11β-HSD1 expression in the human amnion in chorioamnionitis

**Figure 1:**
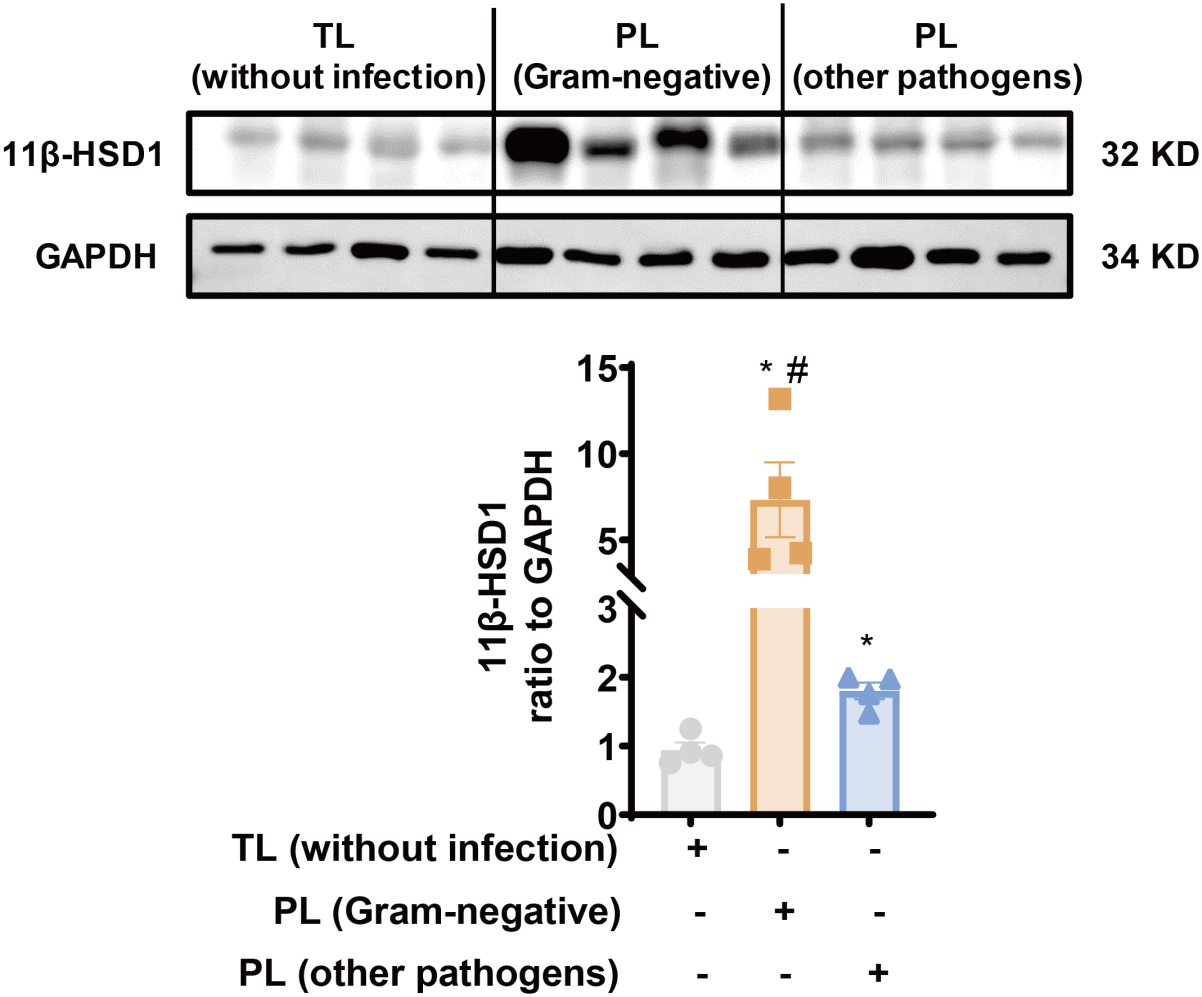
Increased 11β-HSD1 expression in the human amnion in infection-induced chorioamnionitis. Western blotting analysis showing increased 11β-HSD1 levels in the human amnion in either PL with Gram-negative bacterial infection (n=4) or PL with other pathogen infection (n=4) as compared to TL without infection (n=4). A more pronounced increase was observed in PL with Gram-negative bacterial infection. Data are mean ± SEM. Statistical analysis was performed with one-way ANOVA test followed by Newman-Keuls test. *p<0.05 vs. TL without infection, #p<0.05 vs. PL (other pathogens).

Previously, we have demonstrated that 11β-HSD1 expression is significantly increased in the human amnion in TL without infection (24, 33). Here, we compared 11β-HSD1 abundance in the amnion tissue between the PL group with infection and the TL group without infection. The demographic and clinical features of recruited subjects for this study were illustrated in **Table 1**. The results showed that 11β-HSD1 abundance was further increased in the human amnion in preterm birth with either Gram-negative bacteria or other pathogen infection when compared with that in normal spontaneous labor at term without infection (**Figure 1**). Further analysis showed that the increase was more pronounced in infection with Gram-negative bacteria than infection with other microorganisms (**Figure 1**). Detailed information on the pathogen detected in preterm birth was shown in **Supplementary Table 3**. Since the expression of 11β-HSD1 increases with gestational age (23), the higher expression of 11β-HSD1 in the amnion in the PL group with infection than the TL group without infection indicates that infection may be a further inducer of 11β-HSD1 expression in addition to the labor process *per se*.

**Table 1:** Demographic and clinical characteristics of recruited pregnant women.

	TL (n=4)	PL (Gram negative) (n=4)	PL (other pathogens) (n=4)
Maternal Age	28.75 ± 0.947	31.50 ± 1.041	28.50 ± 1.041
Gestation (days)	274.5 ± 3.524	239.3 ± 5.297**	234.5 ± 9.350**
Newborn weight (g)	3249 ± 122.2	2184 ± 152.6**	2303 ± 206.5**
WBC count (*109)	9.520 ± 0.788	18.12 ± 0.978***	12.71 ± 0.809*
Neutrophil ratio to WBC (%)	71.30 ± 1.990	84.60 ± 2.276**	83.65 ± 1.689**
CRP (mg/L)	undetected	23.54 ± 9.501	8.807 ± 3.631
Maternal Body temperature (°C)	36.48 ± 0.063	37.55 ± 0.096***	37.28 ± 0.138***

TL, term labor without infection, PL, preterm labor with infection. Data are mean ± SEM. Statistical analysis was performed with one-way ANOVA test followed by Newman-Keuls test. *p<0.05, **p<0.01, ***p<0.001 vs. TL without infection.

### Synergistic induction of 11β-HSD1 expression by LPS and glucocorticoids in human amnion fibroblasts

**Figure 2:**
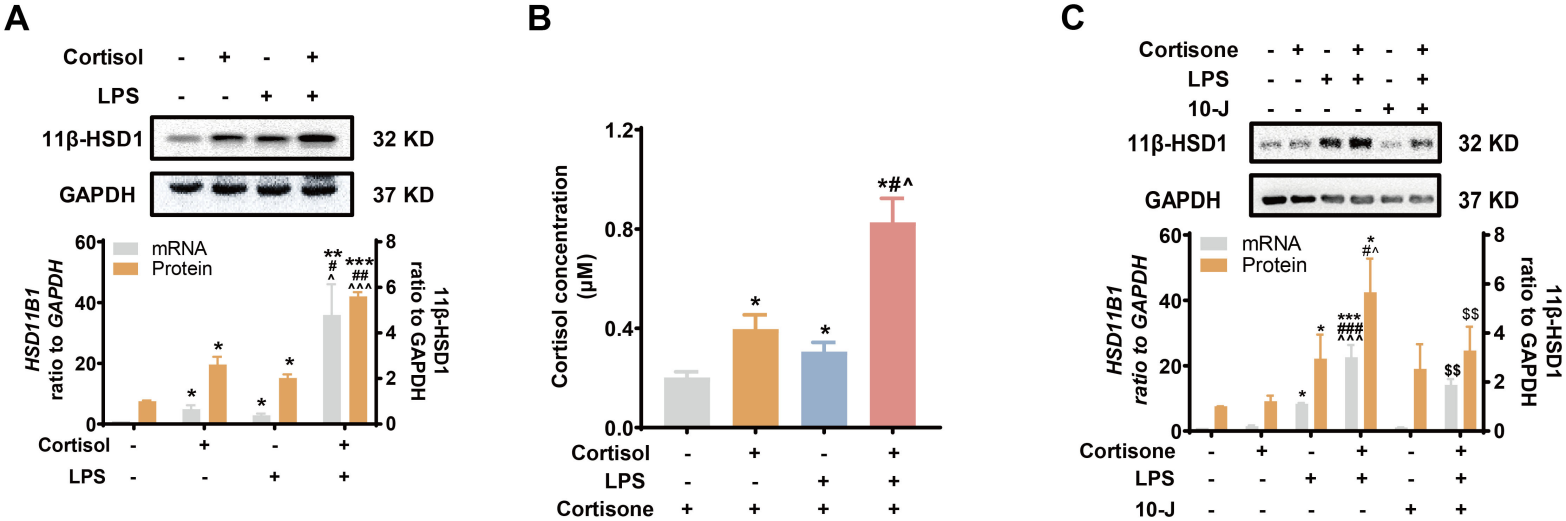
Synergistic induction of 11β-HSD1 expression by LPS and glucocorticoids in human amnion fibroblasts. **(A, B)** Effect of cortisol (1 μM; 24 hours) and LPS (10 ng/mL; 24 hours) on 11β-HSD1 expression and cortisol regeneration (n=4). For the study of cortisol regeneration, the cells were pretreated with cortisol (1 μM) in the presence or absence of LPS (10 ng/mL) for 24 hours. After removal of the treatments by washing, the cells were incubated with cortisone (10 μM), the substrate of 11β-HSD1, for 24 hours. The conditioned culture medium was then collected for cortisol measurement. **(C)** Effect of 11β-HSD1 inhibitor 10-J (1 μM; 24 hours) on the synergistic induction of 11β-HSD1 mRNA (n=4) and protein (n=5) by cortisone (10 μM; 24 hours) and LPS (10 ng/mL; 24 hours). Data are mean ± SEM. Statistical analysis was performed with one-way ANOVA test followed by Newman-Keuls test. *p<0.05, **p<0.01, ***p<0.001 vs. control (without treatment), #p<0.05, ##p<0.01, ###p<0.001 vs. cortisol or cortisone, ^p<0.05, ^^^p<0.001 vs. LPS, $$p<0.01 vs. cortisol or cortisone plus LPS.

Next, we examined whether LPS of the gram-negative bacteria could induce 11β-HSD1 expression synergistically with glucocorticoids. We found that both cortisol (1 μM) and LPS (10 ng/mL) induced 11β-HSD1 mRNA, protein and cortisol regeneration in human amnion fibroblasts (**Figures 2A, B**). Moreover, the induction was synergistic when cortisol and LPS were combined (**Figures 2A, B**). Although cortisone (10 μM) had no effect on 11β-HSD1 mRNA and protein on its own, combination of cortisone and LPS also induced 11β-HSD1 mRNA and protein synergistically, which was significantly attenuated by an 11β-HSD1 inhibitor 10-J (1 μM) (**Figure 2C**), suggesting that the enhancement required the conversion of cortisone to cortisol by 11β-HSD1. These results indicate that further enhancement of cortisol regeneration in preterm birth with infection is ascribed at least in part to the synergistic induction of 11β-HSD1 by LPS and glucocorticoids.

### Effect of glucocorticoids on the induction of COX-2 and pro-inflammatory cytokines by LPS in human amnion fibroblasts

**Figure 3:**
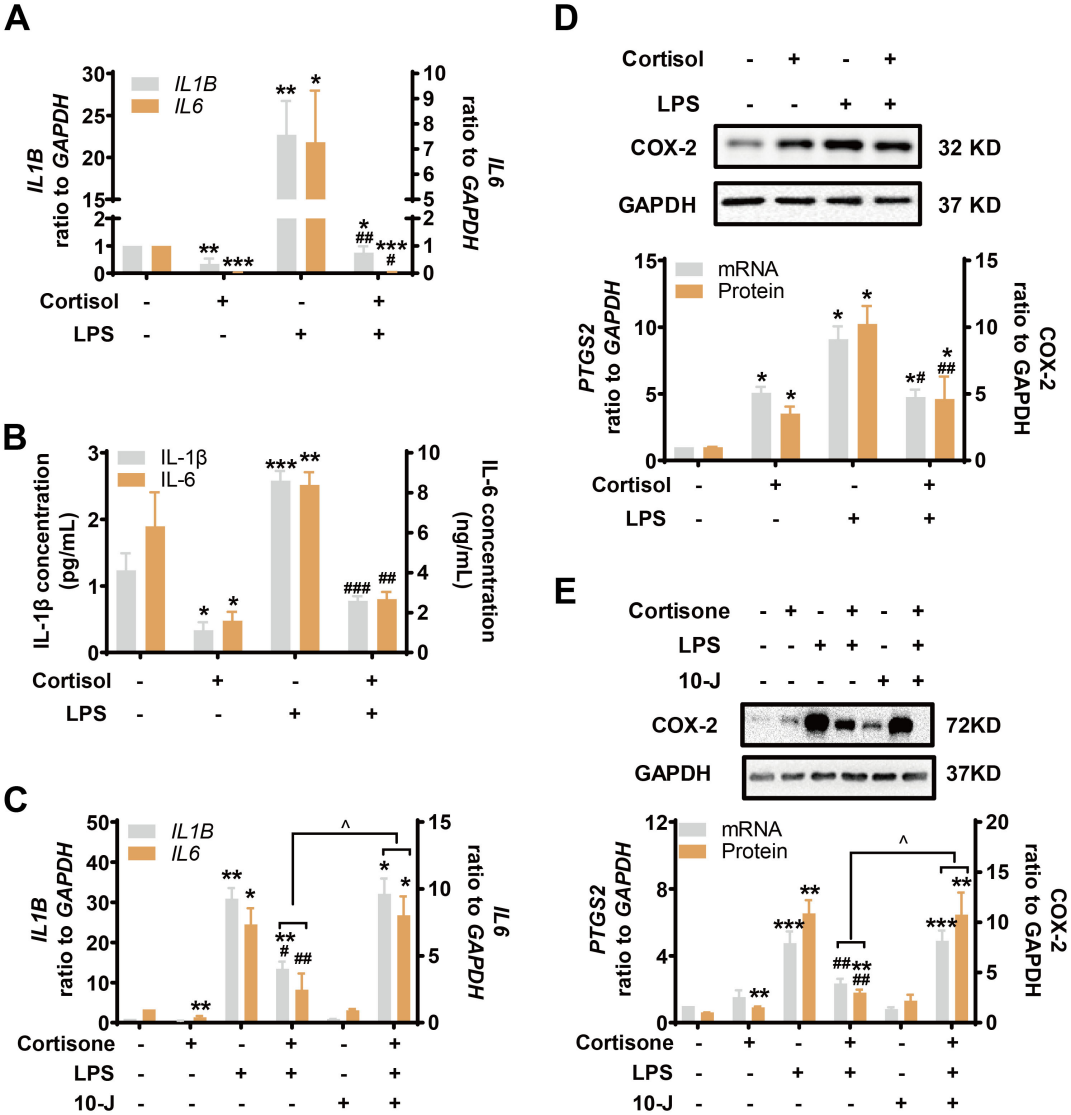
Simultaneous induction of COX-2 and inhibition of pro-inflammatory cytokines by glucocorticoids in the presence and absence of LPS in human amnion fibroblasts. **(A, B)** Effect of cortisol (1 μM; 24 hours) and LPS (10 ng/mL; 24 hours) on IL-1β and IL-6 mRNA expression **(A)** and secretion **(B)** (n=3). **(C)** Effect of cortisone (10 μM; 24 hours), LPS (10 ng/mL; 24 hours) and 11β-HSD1 inhibitor 10-J (1 μM; 24 hours) on IL-1β and IL-6 mRNA (n=4). **(D)** Effect of cortisol (1 μM; 24 hours) and LPS (10 ng/mL; 24 hours) on COX-2 mRNA and protein (n=4). **(E)** Effect of cortisone (10 μM; 24 hours), LPS (10 ng/mL; 24 hours) and 11β-HSD1 inhibitor, 10-J (1 μM; 24 hours) on COX-2 mRNA (n=4) and protein (n=5). Data are mean ± SEM. Statistical analysis was performed with one-way ANOVA test followed by Newman-Keuls test. *p<0.05, **p<0.01, ***p<0.001 vs. control (without treatment), #p<0.05, ##p<0.01, ###p<0.001 vs. LPS, ^p<0.05 vs cortisone plus LPS.

LPS (10 ng/mL) induced both pro-inflammatory cytokines (IL-1β and IL-6) and COX-2 drastically in amnion fibroblasts (**Figures 3A–E**), both of which were inhibited by cortisol (1 μM) (**Figures 3A, B, D**) or cortisone (10 μM) (**Figures 3C, E**). The inhibition by cortisone could be blocked by 10-J (1 μM) (**Figures 3C, E**), indicating again that the effect required the conversion of cortisone to cortisol by 11β-HSD1. Cortisol (1 μM) or cortisone (10 μM) alone significantly decreased pro-inflammatory cytokines (IL-1β and IL-6) but increased COX-2 levels (**Figures 3A–E**) though the effect of cortisone was not as great as that of cortisol (**Figures 3C, E**). Notably, although combination of LPS with cortisol or cortisone significantly suppressed the induction of COX-2 by LPS, the suppression was incomplete, and the remaining increase was about the same level as the induction by cortisol or cortisone alone (**Figures 3D, E**). However, the same concentration of cortisol or cortisone completely blocked the induction of pro-inflammatory cytokines by LPS (**Figures 3A–C**). These results suggest that a differential mechanism may exist in the induction of COX-2 by LPS and cortisol in human amnion fibroblasts.

### Role of the TLR-4/NF-κB and STAT3 pathways in the effects of LPS and glucocorticoids in human amnion fibroblasts

**Figure 4:**
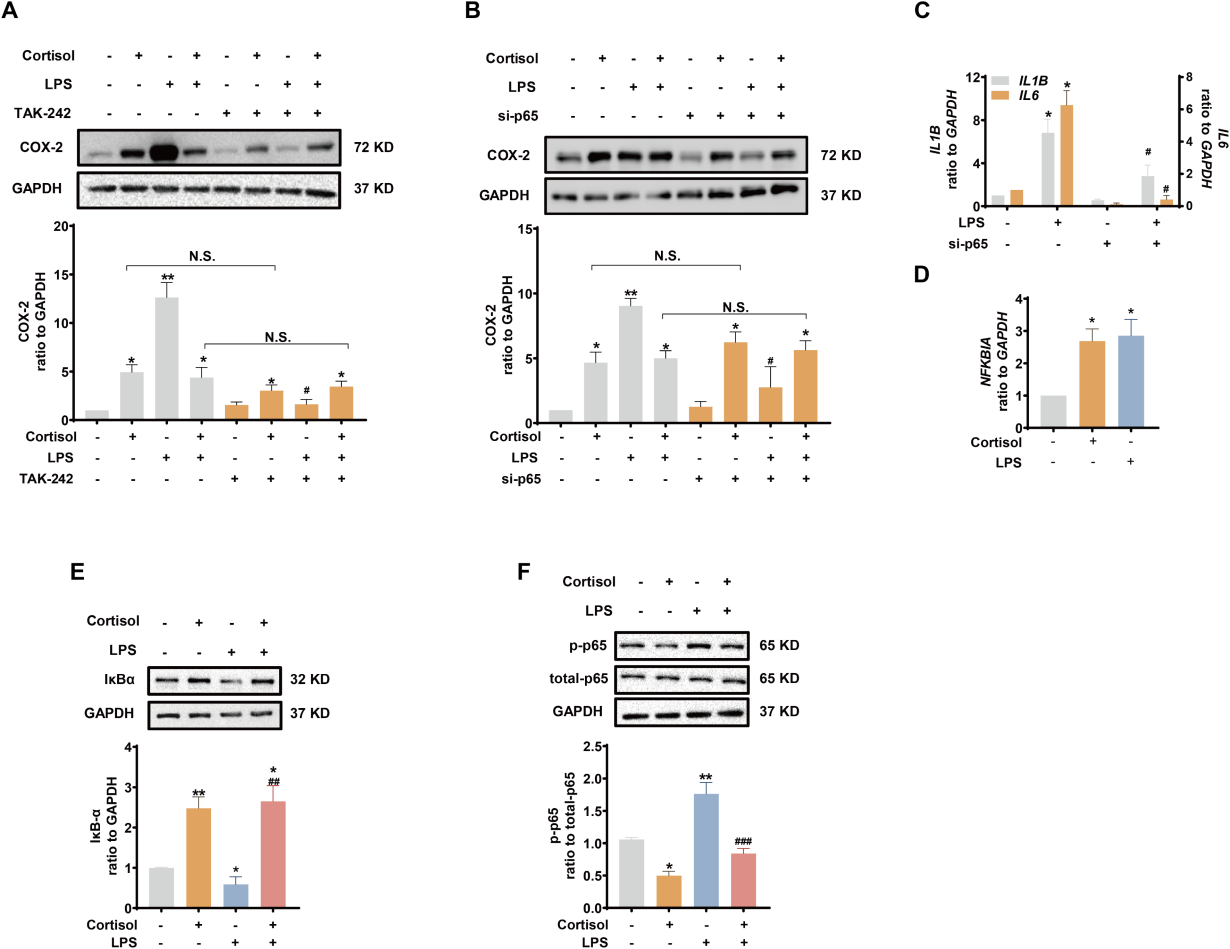
Involvement of NF-κB in the attenuation of LPS-induced pro-inflammatory cytokines and COX-2 expression by glucocorticoids in human amnion fibroblasts. **(A, B)** Effect of TLR-4 inhibitor TAK-242 (10 μM; 24 hours) (A, n=4) and siRNA-mediated knockdown of p65 (si-p65) (B, n=3) on the induction of COX-2 by cortisol (1 μM; 24 hours) and LPS (10 ng/mL; 24 hours). **(C)** Effect of si-p65 on the induction of *IL1B* and *IL6* mRNA by LPS (10 ng/mL; 24 hours) (n=4). **(D, E)** Effect of cortisol (1 μM; 24 hours) and LPS (10 ng/mL; 24 hours) on *NFKBIA* mRNA **(D)** and IκB-α protein **(E)** abundance (n=3). **(F)** Effect of cortisol (1 μM; 3 hours) and LPS (10 ng/mL; 3 hours) on p65 phosphorylation at ser536 (p-p65) (n=3). Data are mean ± SEM. Statistical analysis was performed with one-way ANOVA test followed by Newman-Keuls s test. *p<0.05, **p<0.01 vs. control (without treatment), #p<0.05, ##p<0.01, ###p<0.001 vs. LPS. N.S., none significance.

**Figure 5:**
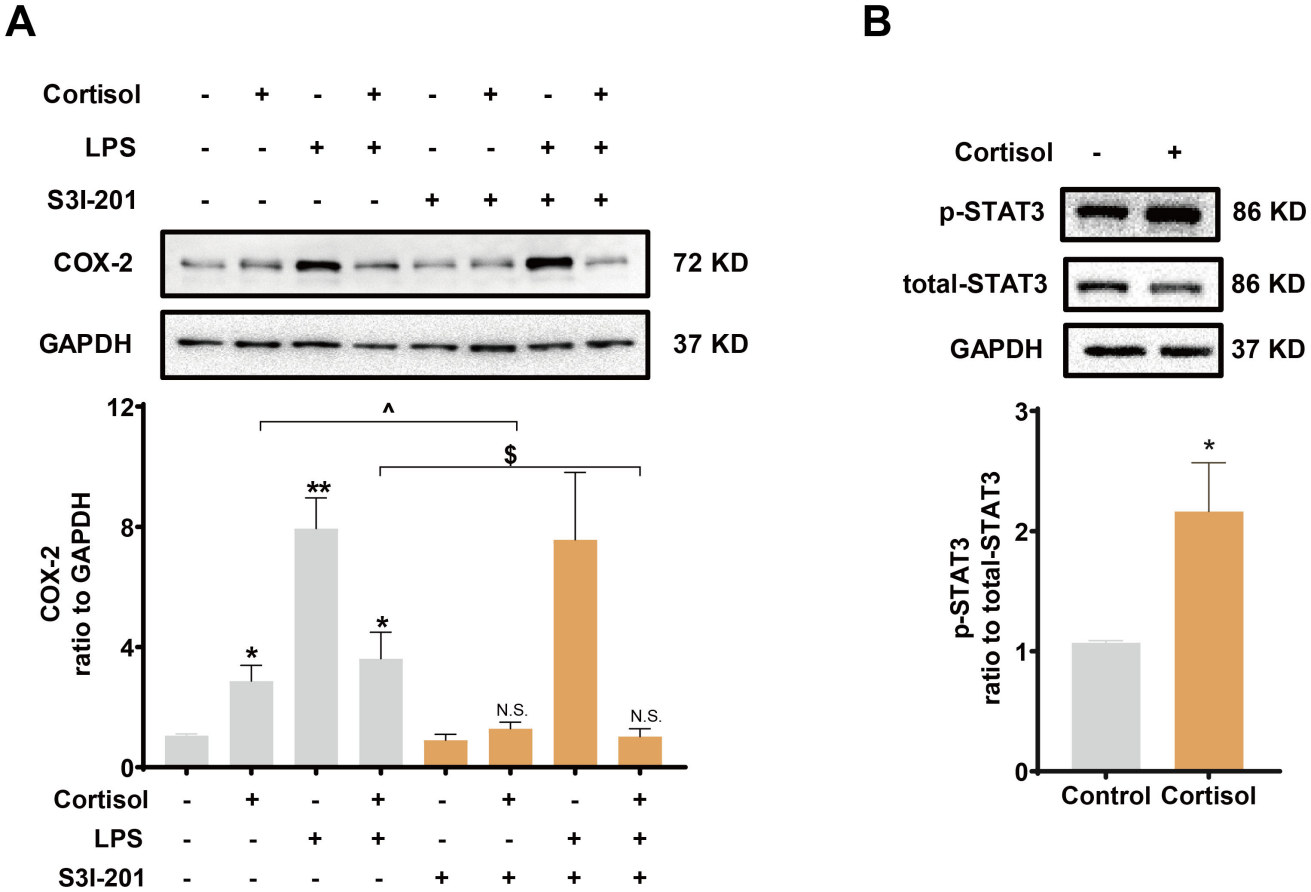
Role of STAT3 in the induction of COX-2 by cortisol in the presence or absence of LPS in human amnion fibroblasts. **(A)** Effect of STAT3 inhibitor S3I-201 (50 μM; 24 hours; n=5) on the induction of COX-2 by cortisol (1 μM; 24 hours) and LPS (10 ng/mL; 24 hours). **(B)** Effect of cortisol (1 μM; 3 hours) on STAT3 phosphorylation at Tyr705 (p-STAT3) (n=4). Data are mean ± SEM. Statistical analysis was performed with one-way ANOVA test followed by Newman-Keuls test **(A)** or paired Student’s t-test **(B)**. *p<0.05, vs. control (without treatment), ^p<0.05, $p<0.05 between lined groups, N.S., none significance vs. control.

Inhibition of TLR-4, the receptor mediating the effect of LPS, with TAK-242 (10 μM) blocked the induction of COX-2 and pro-inflammatory cytokines by LPS (10 ng/mL), but not the induction of COX-2 by cortisol alone (1 μM) or the remaining induction of COX-2 by the combination of LPS and cortisol in human amnion fibroblasts (**Figure 4A**). NF-κB, which is normally sequestered by IκB protein in the cytoplasm, plays a central role in the mediation of infection-induced inflammation upon liberation from IκB (40). Small interference RNA-mediated knock-down of the p65 subunit of NF-κB significantly inhibited the induction of pro-inflammatory cytokines (**Figure 4C**) and COX-2 by LPS (10 ng/mL), but not the induction of COX-2 by cortisol (1 μM) (**Figure 4B**). Of interest, both cortisol (1 μM) and LPS (10 ng/mL) increased *NFKBIA* (encoding IκB-α) mRNA in human amnion fibroblasts (**Figure 4D**). However, cortisol (1 μM) increased while LPS (10 ng/mL) decreased IκB-α protein levels, and the inhibition by LPS could be blocked by cortisol (1 μM) (**Figure 4E**). Moreover, the basal and LPS (10 ng/mL)-induced p65 phosphorylation, an indicator of NF-κB activation, were also inhibited by cortisol (1 μM) (**Figure 4F**). By contrast, inhibition of STAT3 with S3I-201 (50 μM) blocked not only the induction of COX-2 by cortisol alone (1 μM), but also the remaining induction of COX-2 expression with the combination of LPS and cortisol (**Figure 5A**), but not the induction by LPS (10 ng/mL) alone (**Figure 5A**). Consistently, cortisol (1 μM) increased STAT3 phosphorylation (**Figure 5B**). These results indicate that the TLR-4/NF-κB pathway mediates the induction of COX-2 and pro-inflammatory cytokines by LPS, and STAT3 pathway mediates the induction of COX-2 by cortisol alone. The latter may account for the remaining induction of COX-2 with the combination of cortisol and LPS.

### Corticosterone ameliorates fetal organ injury induced by LPS in pregnant mice

**Figure 6:**
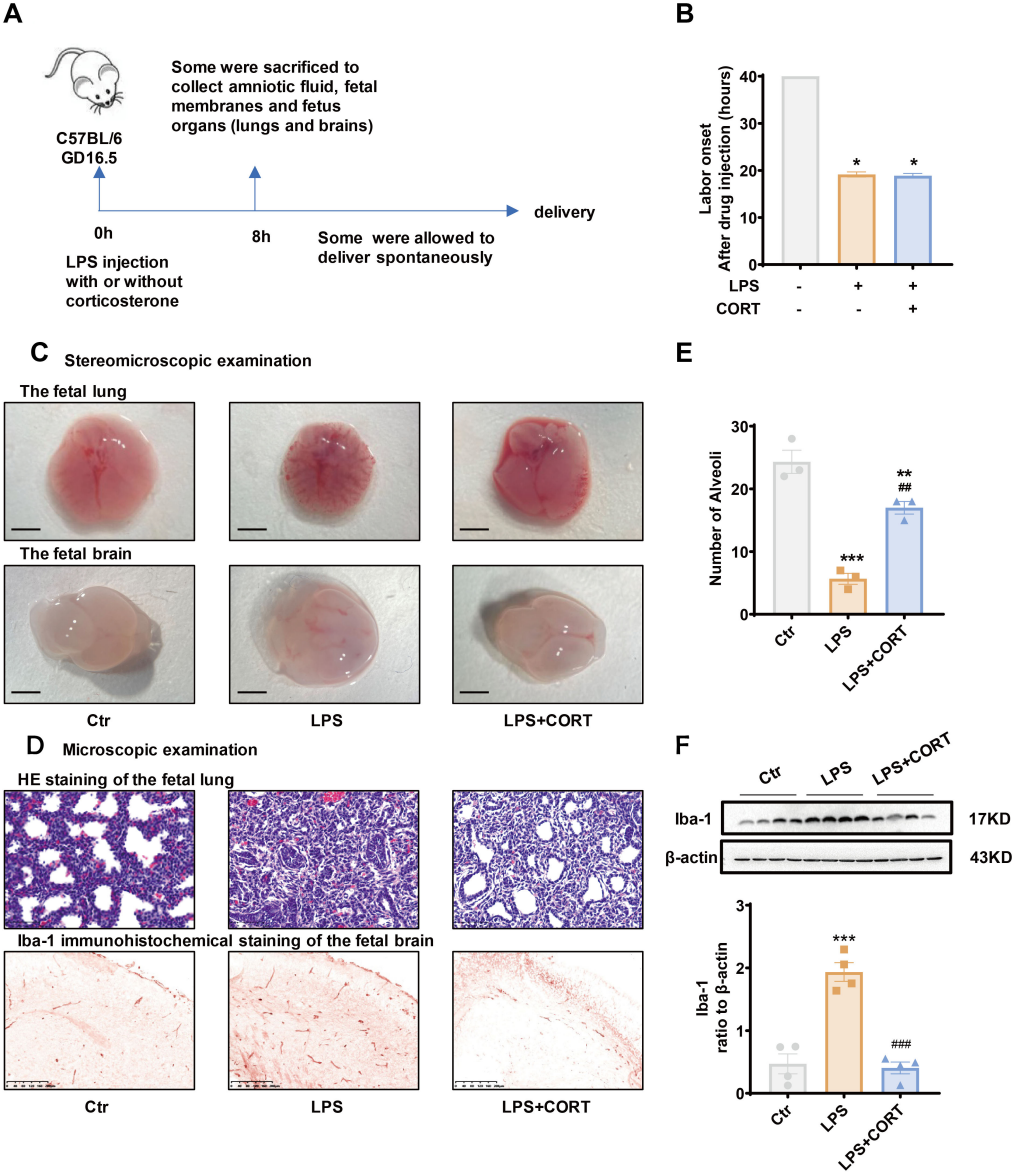
Effect of corticosterone on LPS-induced preterm birth and fetal organ damage in pregnant mice. **(A)** Diagram illustrating experiment procedure in pregnant mice. **(B)** Effect of corticosterone (1 μg per dam) and LPS (20 μg per dam) on the onset of labor. **(C)** Stereomicroscopic examination of the fetal lung and brain following injection of LPS (20 μg per dam) and corticosterone (1 μg per dam). Scale bars, 3mm. **(D)** Microscopic examination of the fetal lung with hematoxylin-eosin (HE) staining and the fetal brain with Iba-1 immunohistochemical staining following injection of LPS (20 μg per dam) and corticosterone (1 μg per dam). Scale bars, 200μm. **(E)** Number of alveoli per field of view in the fetal lung with HE staining (n =3). **(F)** Western blotting showing the effect of LPS (20 μg per dam) and corticosterone (1 μg per dam) on Iba-1 abundance in the fetal brain (n =4). GD, gestational days. CORT, corticosterone. Data are mean ± SEM. Statistical analysis was performed with one-way ANOVA test followed by Newman-Keuls test. *p<0.05, **p<0.01, ***P<0.001 vs. control (without treatment), ##p<0.01, ###p<0.001 vs. LPS.

LPS (20 μg per dam) with or without corticosterone (1 μg per dam) was administered intraperitoneally to pregnant mice on GD 16.5 to mimic intrauterine Gram-negative bacteria infection with or without glucocorticoid treatment (**Figure 6A**). Preterm delivery occurred in all mice about 20 hours after LPS injection (**Figure 6B**). Corticosterone co-treatment failed to change the course of preterm birth induced by LPS (**Figure 6B**), but alleviated LPS-induced fetal organ damages (**Figures 6C–F**). Stereomicroscopic examination of the fetal lung and brain showed that LPS caused bleeding and edema in both organs (**Figure 6C**). Microscopic examination of HE-stained fetal lung section showed a reduction in alveoli along with collapsed bronchial wall with LPS administration (**Figures 6D, E**). Immunohistochemical staining and Western blotting showed increased Iba-1 abundance, a marker of brain response to injury (51–53), in the fetal brain with LPS administration (**Figures 6D, F**). Corticosterone co-treatment alleviated those lesions and the increase in Iba-1 caused by LPS (**Figures 6C–F**).

**Figure 7:**
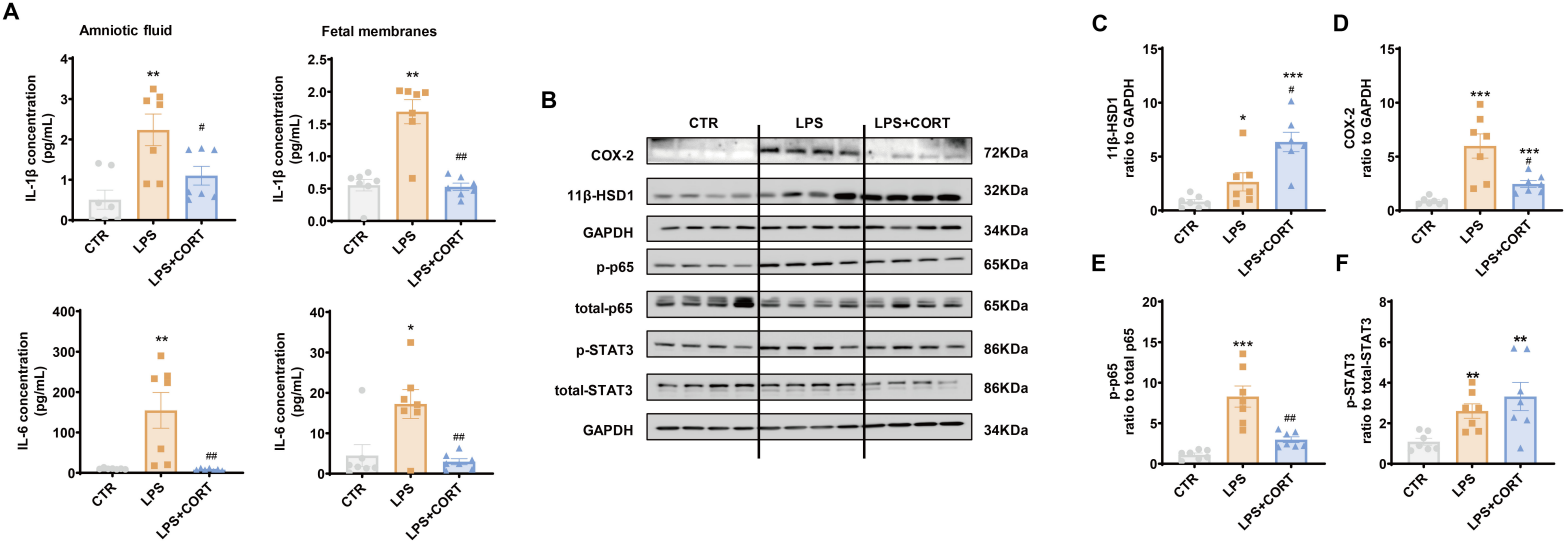
Effect of LPS and corticosterone injection on the abundance of pro-inflammatory cytokines, COX-2, 11β-HSD1, p-p65 and p-STAT3 in mouse intrauterine tissues. **(A)** Effect of LPS (20 μg per dam) and corticosterone (1 μg per dam) injection on IL-1β and IL-6 abundance in the amnion fluid and fetal membranes (n = 7 each group). **(B–F)** Effect of LPS (20 μg per dam) and corticosterone (1 μg per dam) injection on 11β-HSD1, COX-2, p65 phosphorylation at Ser536 (p-p65) and STAT3 phosphorylation at Tyr705 (p-STAT3) in the fetal membranes (n=7 each group). Data are mean ± SEM. Statistical analysis was performed with one-way ANOVA test followed by Newman-Keuls test. *p<0.05, **p<0.01, ***P<0.001 vs. control (without treatment), #p<0.05, ##p<0.01 vs. LPS.

ELISA showed that increases in IL-1β and IL-6 in the amniotic fluid and fetal membranes induced by LPS were blocked by corticosterone co-treatment (**Figure 7A**). By contrast, the increase in 11β-HSD1 induced by LPS in the fetal membranes was enhanced by corticosterone co-treatment (**Figures 7B, C**). Although corticosterone co-treatment also suppressed the induction of COX-2 by LPS in the fetal membranes, only partial suppression was observed (**Figures 7B, D**). The remaining COX-2 induction with LPS and corticosterone co-treatment was still significant compared to the control group, which may be attributed to the induction by corticosterone *per se*. In line with the notion, the induction of p65 phosphorylation in the fetal membranes that mediates the induction of pro-inflammatory cytokine and COX-2 expression by LPS, was completely blocked by corticosterone co-treatment, while STAT3 phosphorylation, a major mediator of COX-2 expression by glucocorticoids, remained in the LPS and corticosteroid co-treatment group (**Figures 7B, E, F**).

## Discussion

The inner amnion layer of the fetal membranes, being the most resilient layer, plays a vital role in maintaining the integrity of the fetal membranes and sterile intrauterine environment. Beyond its physical barrier function, the amnion is also a source of a variety of bioactive substances that contribute to parturition and immunomodulation (20, 54, 55). In this study, we demonstrated that increased glucocorticoid regeneration by 11β-HSD1 in the amnion layer of the fetal membranes might play a dual role in parturition. On the one hand, glucocorticoid accumulation may promote parturition through stimulation of prostaglandin synthesis via induction of COX-2 expression by a NF-κB-independent pathway such as STAT3 as demonstrated in this study as well as in previous studies (24, 26, 37–39). On the other hand, glucocorticoids may also restrain the production of pro-inflammatory cytokines through a NF-κB-dependent pathway to avoid excessive pro-inflammatory cytokine production at parturition. Of interest, we found that 11β-HSD1 expression could be further enhanced when infection was present, particularly in Gram-negative bacteria infection. LPS of the Gram-negative bacteria could induce 11β-HSD1 expression synergistically with glucocorticoids. These results indicate that the pro-parturition and anti-inflammatory actions of glucocorticoids in the fetal membranes may be further strengthened when infection is present. We believe that this synergistic enhancement of glucocorticoid regeneration is a self-protective measure of the fetus threatened by *in-utero* infection so that brakes can be applied in time to prevent excessive pro-inflammatory cytokine production while not affecting the timing of labor for the safety of the fetus.

In addition to infection-induced chorioamnionitis, sterile inflammation also exists in the fetal membranes in normal parturition with increased expression of pro-inflammatory cytokines including IL-1β and TNF-α (56). Our previous studies have demonstrated that these pro-inflammatory cytokines can synergize with cortisol to induce 11β-HSD1 expression in the fetal membranes as well (28, 30), indicating that cortisol regeneration may also play a dual role in in normal parturition.

Our previous studies have shown that the induction of COX-2 by glucocorticoids in human amnion fibroblasts involves the interaction of GR with a myriad of transcription factors including STAT3, CREB, and C/EBPδ (24, 26, 57). In this study, we demonstrated again that STAT3 was involved in the induction of COX-2 by glucocorticoids in human amnion fibroblasts, and inhibition of STAT3 blocked the induction of COX-2 by glucocorticoids either on its own or in combination with LPS. Because inhibition of STAT3 was unable to block the induction of COX-2 by LPS, we believe that the remaining induction of COX-2 with the combination of cortisol and LPS is due to the induction by cortisol *per se* through a NF-κB-independent pathway rather than due to the incomplete blockade of LPS-induced COX-2 by cortisol. These NF-κB-independent labor-inducing actions of glucocorticoids may remain to help the fetus deliver in time so that detrimental effects of excessive pro-inflammatory cytokines on the fetus can be avoided at parturition. Together with the well-described effects of glucocorticoids on fetal organ maturation (58–60), these actions of glucocorticoids would increase the likelihood of fetal survival when infection-induced preterm birth is inevitable.

In addition to COX-2 induction, glucocorticoid regeneration may also promote membrane rupture at parturition by inhibiting collagen cross-linking, inducing collagen cleavage via an NF-κB-independent pathway in human fetal membranes (32–34). Moreover, glucocorticoids may also promote parturition by stimulating estrogen synthesis and progesterone withdrawal via induction of aromatase and 20α-hydroxysteroid dehydrogenase (20α-HSD) expression in human placenta and fetal membranes respectively (25, 61, 62). These pro-parturition effects of glucocorticoids may offset the parturition-delaying effect brought on by the anti-inflammatory effect of glucocorticoids, which may explain why preterm birth still occurred in the dam that received co-administration of LPS and corticosterone in this study. However, the LPS-induced damage on the fetal organs was greatly alleviated by corticosterone treatment, which is more likely to be a benefit of the anti-inflammatory action of glucocorticoids either regenerated endogenously in intrauterine tissues or from glucocorticoid treatment.

As we demonstrated previously, cortisol not only induced prostaglandin synthesis but also ECM remodeling and progesterone withdrawal in the fetal membranes (25, 32–34, 37–39). Although cortisol regeneration in the fetal membranes has been shown to be implicated in these pivotal events of parturition, it is intriguing that systemic administration of glucocorticoids, particularly dexamethasone, often fails to affect gestational age, which may be attributed to the potent negative feedback effects of systemic administration of glucocorticoids on maternal and fetal hypothalamus-pituitary-adrenal axes. These negative feedback effects may obscure the pro-laboring effects of endogenous steroids synthesized by the maternal and fetal adrenals. However, these complicated effects of systemic administration of glucocorticoids do not necessarily mean that cortisol regenerated locally in the fetal membranes plays no role in parturition. As a matter of fact, clinical observations showed that intra-amniotic injection of glucocorticoids indeed shortened gestational age (63–66).

In addition to glucocorticoids, there are other factors such as galectin-1, prolactin, progesterone, and IL-10 have been reported to inhibit infection-induced inflammatory responses in the intrauterine tissues (67–70). It would be of interest to investigate the interaction of cortisol with these factors in the fetal membranes. In patients with nasal polyps, glucocorticoid treatment has been shown to enhance galectin-1 expression in their surgical resection (71). We are not sure whether this interaction also exists in the fetal membranes. In the case of progesterone, the situation is more complicated as progesterone also carries other pregnancy-maintaining actions in addition to its anti-inflammatory actions. In the fetal membranes, cortisol has been shown to induce progesterone functional withdrawal by upregulating its degradation enzyme 20α-HSD (25).

**Figure 8:**
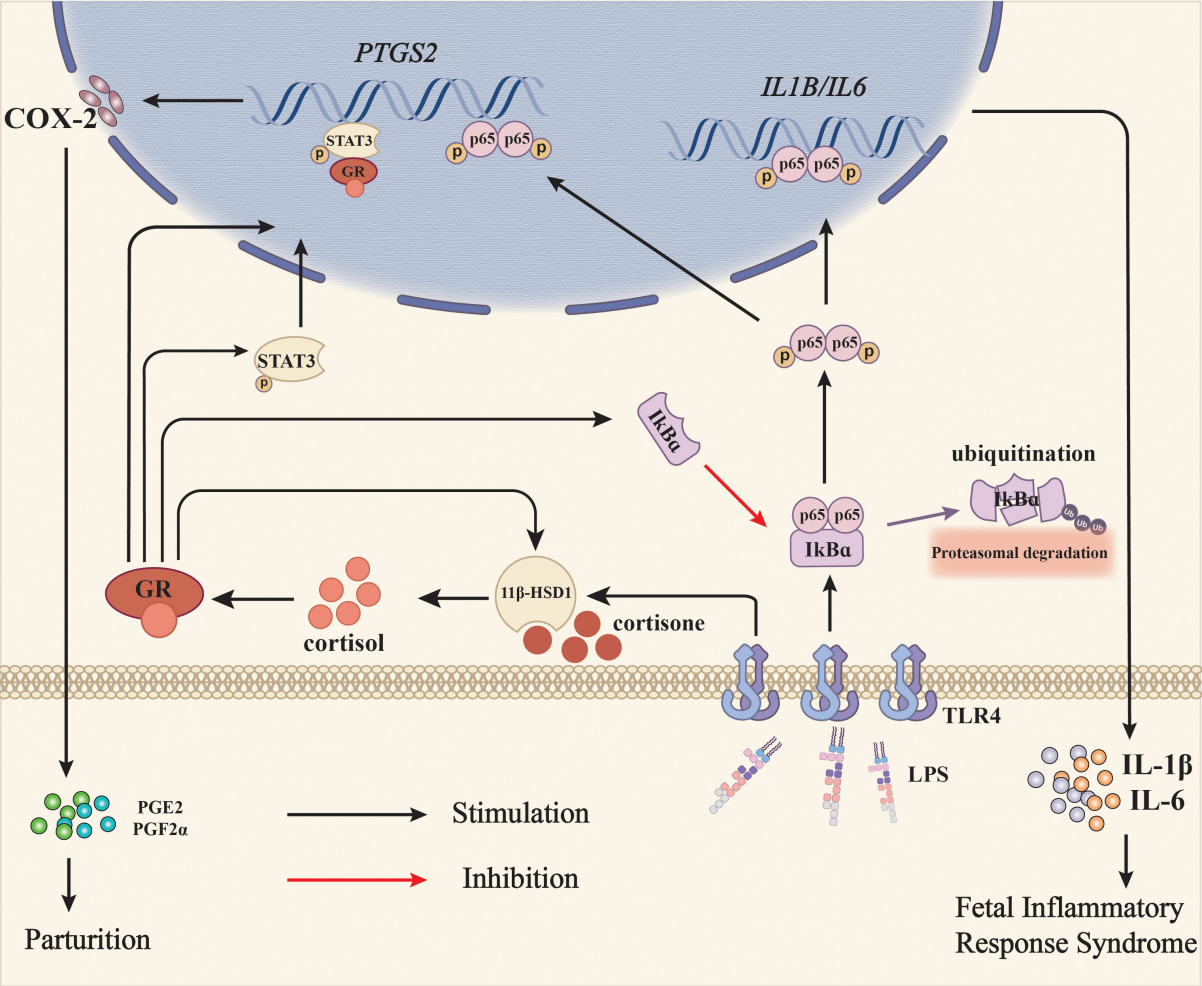
Diagram illustrating the role of glucocorticoid regeneration of the fetal membranes in parturition with infection-induced chorioamnionitis. When intrauterine infection is present in pregnancy, LPS from Gram-negative bacteria activates NF-κB via the TLR4 receptor, which subsequently induces the expression of pro-inflammatory cytokines such as IL-1β and IL-6, and COX-2, the rate-limiting enzyme in prostaglandin synthesis. Although prostaglandins synthesized by COX-2 are pro-parturition, excessive pro-inflammatory cytokines may harm the fetus and lead to fetal inflammatory response syndrome (FIRS). Meanwhile, LPS also induces 11β-HSD1 expression either by itself or in synergy with glucocorticoids, resulting in increased conversion of biologically inactive cortisone to bioactive cortisol. Cortisol not only suppresses LPS-induced pro-inflammatory cytokine and COX-2 expression via inhibition of NF-κB, but also induces COX-2 expression on its own through NF-κB-independent pathways such as activation of STAT3, thereby minimizing the fetus-hazardous effects of pro-inflammatory cytokines at parturition and helping the fetus escape from the *in-utero* environment affluent in pro-inflammatory cytokines at the same time. Black arrows indicate stimulation and the red arrow indicates inhibition.

In summary, our study illustrates that glucocorticoid regeneration is implicated in both pro-parturition and anti-inflammatory actions in the fetal membranes, which may minimize fetus-hazardous effects imposed by pro-inflammatory cytokines at parturition (**Figure 8**), and help the fetus escape from the *in-utero* environment affluent in pro-inflammatory cytokines at parturition. This dual role of glucocorticoid regeneration in the fetal membranes may be of particular importance for the survival of the fetus in infection-induced chorioamnionitis. However, it should be kept in mind that over inhibition of the immune responses by glucocorticoids may also exacerbate bacterial infection. For the safety and survival of the preterm baby under conditions of chorioamnionitis, it is important to deliver the baby timely with functional lungs. In consideration of the pro-laboring and lung maturing effects of glucocorticoids, it is may be beneficial to give glucocorticoids along with adequate antibiotics when the fetus is endangered by intrauterine infection. Therefore, it presents a tricky task to balance these effects of glucocorticoids in infection-induced chorioamnionitis in the future.

## Data Availability

The original contributions presented in the study are included in the article/**Supplementary Material**. Further inquiries can be directed to the corresponding authors.
