# Supplementary material for: The dual role of glucocorticoid regeneration in inflammation at parturition
Source: Front Immunol. 2024 Sep 3;15:1459489. doi: 10.3389/fimmu.2024.1459489 (PMC11405189; doi:10.3389/fimmu.2024.1459489)
Supplement: Supplementary file 1 [file DataSheet1.docx]

**The dual role of glucocorticoid regeneration in inflammation at parturition**

Li-Jun Ling^1#^, Qiong Zhou^2#^, Fan Zhang^3,4^, Wen-Jia Lei^3,4^, Meng-Die Li^3,4^, Jiang-Wen Lu^3,4^, Wang-Sheng Wang^3,4*^, Kang Sun^3,4*^, Hao Ying^5,6,7*^


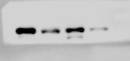

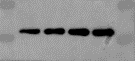


**total-p65**

**65 KD**

**GAPDH**

**37 KD**

**p65 siRNA**

**Scrambled siRNA**

**Supplementary Figure 1. Knock-down efficiency of p65.** N=3. Data are mean ± SEM. Statistical analysis was performed with paired Student’s t-test. ***p<0.001 vs. scrambled siRNA (negative control, NC).

**Supplementary Table 1. Primer sequences used for qRT-PCR.**

| Gene | Forward sequences | Reverse sequences |
| --- | --- | --- |
| *HSD11B1* | GCAGCCTCAGCACACTACAT | CATGTCTAGTCCTCCCATGAGC |
| *PTGS2* | TGTGCAACACTTGAGTGGCT | ACTTTCTGTACTGCGGGTG |
| *IL1B* | CCACAGACCTTCCAGGAGAATG | GTGCAGTTCAGTGATCGTACAGG |
| *IL6* | ACTCACCTCTTCAGAACGAATTG | CCATCTTTGGAAGGTTCAGGTTG |
| *NFKBIA* | GGGCTATTCTCCCTACCAGC | TCATCATAGGGCAGCTCGTC |
| *GAPDH* | CCCCTCTGCTGATGCCCCCA | TGACCTTGGCCAGGGGTGCT |

**Supplementary Table 2. Antibodies used in this study.**

| Protein | Application | Dilution ratio | Manufacturer | Cat. |
| --- | --- | --- | --- | --- |
| 11β-HSD1 | Western blot | 1:500 | Abcam | ab157223 |
| IκB-α | Western blot | 1:500 | Santa Cruz | sc-4094 |
| COX-2 | Western blot | 1:1000 | Cell Signaling | 12282 |
| total p65 | Western blot | 1:1000 | Cell Signaling | 6956 |
| phosphorylated p65 (ser536) | Western blot | 1:1000 | Cell Signaling | 3033 |
| total STAT3 | Western blot | 1:1000 | Cell Signaling | 9139 |
| phosphorylated STAT3 (tyr 705) | Western blot | 1:1000 | Cell Signaling | 9145 |
| Iba-1 | Western blot | 1:1000 | Cell Signaling | 17198 |
| Iba-1 | immunohistochemistry | 1:100 | Cell Signaling | 17198 |

**Supplementary Table 3. Information on pathogens in preterm labor with infection (PL)**

| Samples | Pathogens |
| --- | --- |
| PL with Gram-negative bacteria |  |
| Sample 1 | *Klebsiella pneumoniae* |
| Sample 2 | Bacteria  (Gram-negative, not defined) |
| Sample 3 | *Klebsiella pneumoniae* |
| Sample 4 | *Escherichia coli* |
| PL with other pathogens |  |
| Sample 1 | Fungus |
| Sample 2 | *Ureaplasma urealyticum* |
| Sample 3 | *Candida albicans* |
| Sample 4 | *Candida albicans* |
